# Aptamer-Functionalized Liposomes for Targeted Delivery of Anticancer Drugs in Lung Cancer

**DOI:** 10.3390/molecules31173134

**Published:** 2026-09-07

**Authors:** Daniela Leitão, David Moreira, Jéssica Lopes-Nunes, Joana Figueiredo, Carla Cruz

**Affiliations:** RISE-Health UBI, Department of Chemistry, Faculty of Sciences, University of Beira Interior, Rua Marquês d’Ávila e Bolama, 6201-001 Covilhã, Portugaldavid.moreira@ubi.pt (D.M.); jessicalonu@hotmail.com (J.L.-N.);

**Keywords:** lung cancer, G-quadruplex aptamers, nucleolin, liposomes, targeted therapy

## Abstract

Aptamer-functionalized liposomes are a promising strategy to improve the selectivity of anticancer therapies. AT11-L2 is a G-quadruplex (G4)-forming aptamer with high affinity for nucleolin (NCL), which is overexpressed at the surface of non-small cell lung cancer (NSCLC) cells. Here, we developed AT11-L2-functionalized liposomes loaded with doxorubicin (DOX) or BRACO-19 for targeted delivery to NSCLC cells. The effects of both compounds on AT11-L2 stability and the ability of the aptamer to retain G4 folding after liposome conjugation were evaluated. Liposomes were characterized for size, polydispersity, surface charge, stability, encapsulation efficiency, and release profile. Biological activity was assessed in A549 and MRC-5 cells. Liposomes had an average size of approximately 110 nm, with a slight size increase and reduced surface charge after AT11-L2 functionalization. The aptamer retained G4 folding in 100 mM KCl after conjugation. Encapsulation efficiency was approximately 90%. DOX showed substantial release within 72 h, whereas BRACO-19 exhibited a more sustained profile. AT11-L2-DOX liposomes showed preferential effects in A549 cells, while BRACO-19 formulations displayed lower selectivity and cytotoxicity. Additionally, the NCL-dependent internalization of AT11-L2-functionalized liposomes was supported by a protein-blocking assay using an anti-NCL antibody. These results support AT11-L2-functionalized liposomes as a versatile NCL-targeted delivery system for NSCLC.

## 1. Introduction

Lung cancer (LC) is the leading cause of oncological disease-related mortality worldwide, accounting for approximately 19% of all cancer deaths [1,2]. Histologically, LC can be classified into small-cell lung cancer (SCLC) and non-small cell lung cancer (NSCLC), the latter being divided into adenocarcinoma, squamous cell carcinoma, and large-cell carcinoma. NSCLC is the most prevalent type of LC, accounting for 85% of clinical cases [3]. Treatment strategies for NSCLC depend on the stage of the disease and can include surgery (lobectomy or pneumectomy), chemotherapy, radiotherapy or immunotherapy, or a combination thereof [4]. However, because most patients are diagnosed at advanced stages (III and IV), only a small percentage are eligible for surgical resection [5]. Despite substantial advances in systemic therapies, current treatments still face several limitations, including drug resistance, systemic toxicity, and poor selectivity toward tumor cells [6,7]. To overcome these challenges, alternatives that are selective and non-toxic to non-malignant cells have been investigated. Among these, aptamer-based nanoparticles have emerged to protect and deliver drugs to NSCLC cells [5].

Aptamers are short sequences of single-stranded ribonucleic acid (RNA) or deoxyribonucleic acid (DNA) that bind with high affinity and specificity to their targets. Although often referred to as “chemical antibodies”, aptamers offer several advantages over antibodies, including rapid synthesis, low production costs, high stability, low toxicity, and immunogenicity [8,9]. Aptamers with guanine-rich sequences can fold into G4 structures, and in some cases, adopting a secondary structure is essential for their biological activity and target recognition, making them promising therapeutic agents in LC [10]. G4 aptamers have several advantages over unstructured oligonucleotides, as they are thermodynamically and chemically stable, resistant to serum nucleases, and have high cellular uptake [11,12]. An example of these aptamers is AS1411, which binds specifically to nucleolin (NCL), a protein highly expressed on the surface of different cancer cell types, particularly NSCLC [12,13]. Accordingly, AS1411 has been evaluated in several clinical trials [14,15]. However, due to its high degree of polymorphism, the biologically active structure of G4 has not yet been reported. Thus, Phan and co-workers identified several derivatives of AS1411, including AT11-L2 [16]. Previously, we demonstrated that AT11-L2 predominantly adopts a parallel G4 topology in the presence of 100 mM KCl and displays high affinity towards NCL [17], highlighting its potential as a targeting moiety for nanomedicine application [8].

Among the different nanoparticles, liposomes are among the most widely used nanocarriers in clinical applications because of their biocompatibility, biodegradability, and ability to encapsulate both hydrophilic and hydrophobic drugs [18,19]. However, conventional liposomes lack intrinsic molecular specificity toward cancer cells, which may limit selective uptake and contribute to off-target exposure. Functionalization of the liposomal surface with aptamers may overcome this limitation by promoting specific molecular recognition and cellular internalization. In this context, conjugation of the G4-forming aptamer AT11-L2 to liposomes is particularly attractive because AT11-L2 binds nucleolin (NCL), a protein overexpressed at the surface of NSCLC cells. Thus, this strategy combines the drug-loading capacity of liposomes with the molecular targeting properties of AT11-L2, with the aim of improving selective delivery to malignant cells while limiting exposure to non-malignant cells.

To evaluate the versatility of this targeted nanoparticle, two anticancer compounds with distinct physicochemical properties and mechanisms of action were selected. DOX is a hydrophilic anthracycline widely used in chemotherapy but is limited by systemic toxicity and poor tumor selectivity [20,21]. In contrast, BRACO-19 is a hydrophobic G4-targeting compound with intrinsic anticancer activity through stabilization of genomic G4 structures, particularly telomeric G4s [22,23]. Importantly, BRACO-19 was selected as a therapeutic cargo rather than as a stabilizer of the AT11-L2 aptamer. In this nanosystem, AT11-L2 primarily acts as an NCL-targeting moiety, whereas BRACO-19 provides a mechanistically distinct G4-targeting therapeutic component. Therefore, the use of DOX and BRACO-19 also allowed us to assess the versatility of AT11-L2-functionalized liposomes for the delivery of compounds with distinct physicochemical properties and mechanisms of action. The novelty of the present study lies in the development of an NCL-targeted liposomal platform based on the G4-forming aptamer AT11-L2 and the evaluation of its versatility for the delivery of anticancer agents. To accomplish this, we first evaluated the effects of DOX and BRACO-19 on AT11-L2 stability. Subsequently, liposomes were prepared, functionalized with AT11-L2, and physicochemically characterized. Encapsulation efficiency and release profiles were also determined. Finally, cellular uptake, NCL-dependent internalization, and cell viability were evaluated in A549 NSCLC cells and non-malignant MRC-5 lung fibroblasts.

## 2. Results and Discussion

**Figure 1:**
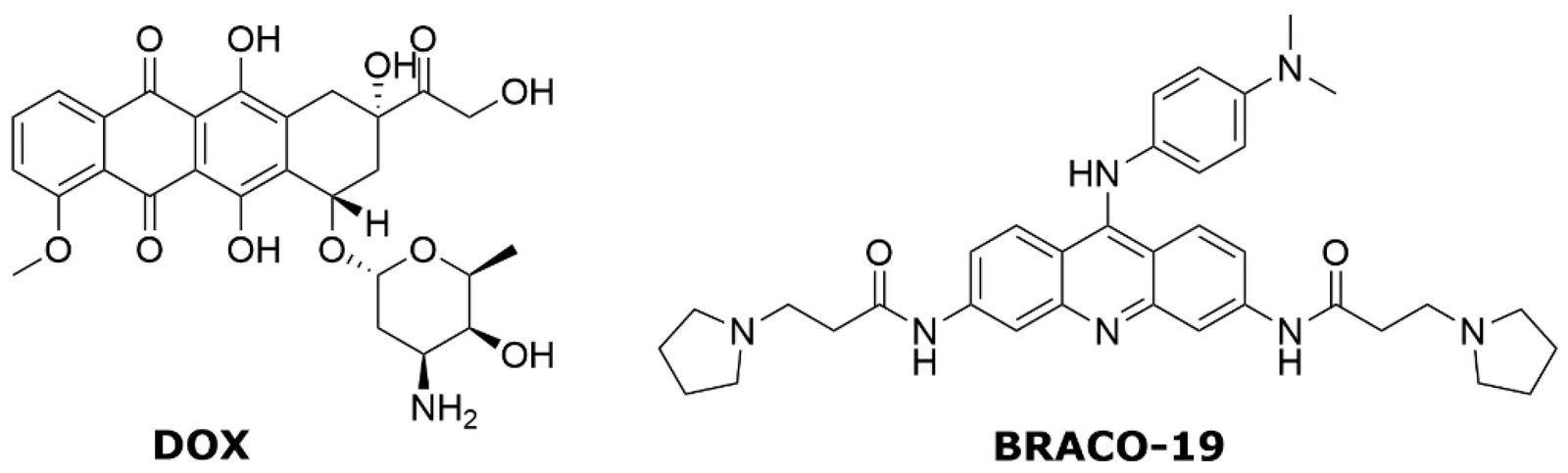
Chemical structures of DOX and BRACO-19 (obtained by ChemDraw 20.0).

Aptamers containing guanine-rich sequences can fold into G4 structures, which can be particularly useful for therapeutic purposes since they allow selective targeting. Previously, we demonstrated that AT11-L2 formed a G4 structure that is capable of binding to NCL with high affinity [17]. Therefore, in the present study, two delivery systems functionalized with AT11-L2 for the selective delivery of DOX and BRACO-19 (Figure 1) to NSCLC cells were developed. DOX and BRACO-19 were selected as two anticancer agents with distinct mechanisms of action and physicochemical properties. DOX is a clinically established hydrophilic anthracycline whose efficacy is limited by systemic toxicity and poor tumor selectivity, making it a suitable model drug for evaluating whether AT11-L2 functionalization can improve the targeting potential of liposomal delivery. In contrast, BRACO-19 is a hydrophobic G4 ligand with intrinsic anticancer activity through stabilization of genomic G4 structures, allowing the versatility of the platform to be assessed with a mechanistically and physicochemically distinct cargo.

Although AT11-L2 and the encapsulated compounds are localized in distinct physicochemical compartments of the final liposomal formulation, the effects of DOX and BRACO-19 on AT11-L2 thermal stability were evaluated as a preliminary structural-compatibility assessment. These experiments were designed to determine whether direct exposure to either compound could markedly disrupt or stabilize the G4 fold of AT11-L2, rather than reproduce the spatial organization of the final liposomal system. Maintenance of the AT11-L2 G4 structure is particularly relevant because this conformation is required for efficient NCL recognition.

**Figure 2:**
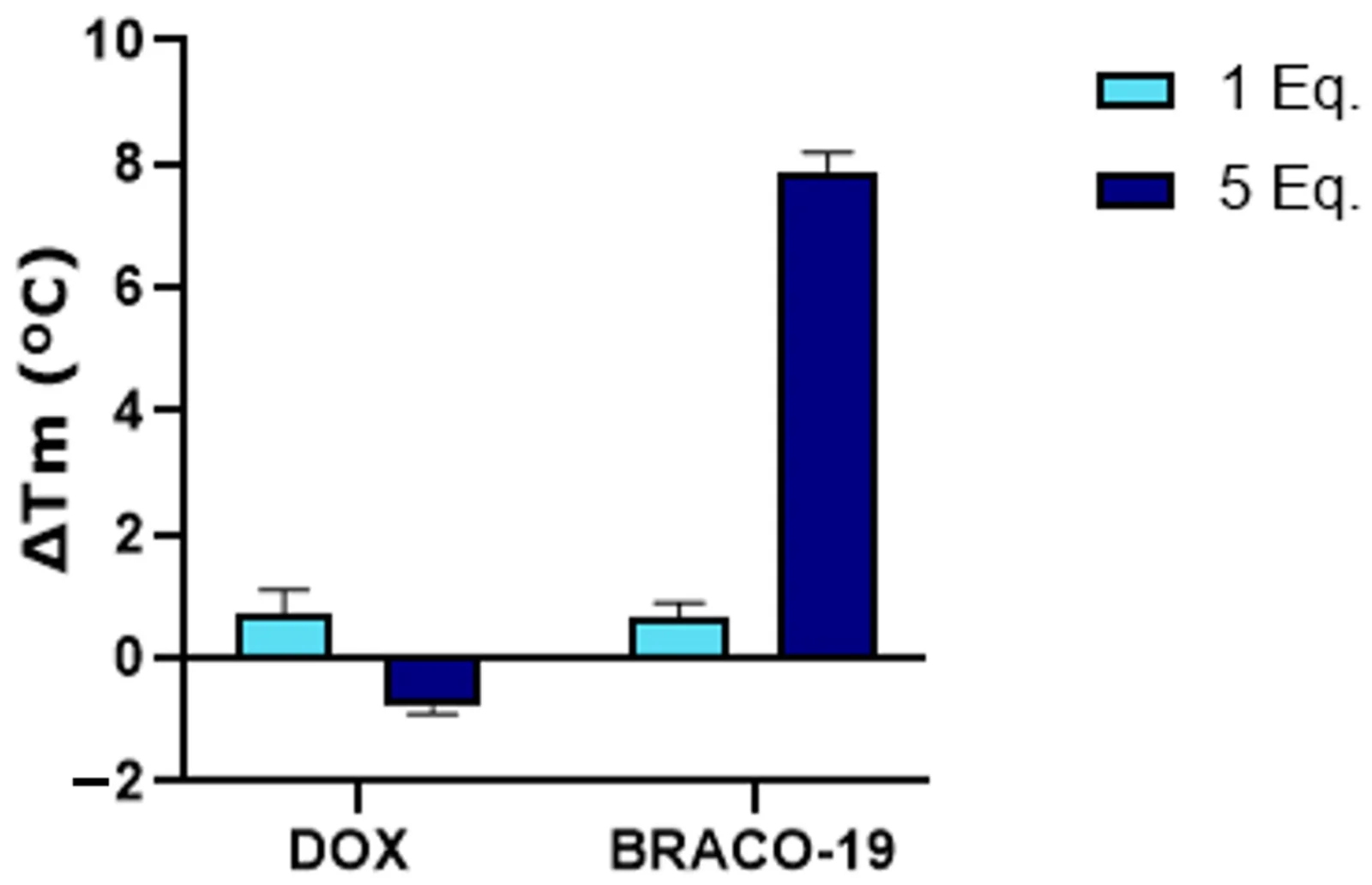
Stabilization of AT11-L2 G4 after the addition of 1 and 5 molar equivalents of DOX and BRACO-19 obtained by FRET-Melting (plotted with OriginPro2021). The results represent the mean ± SD of an experiment (n = 1) performed in duplicate.

Firstly, the ability of BRACO-19 and DOX to stabilize the AT11-L2 G4 structure was evaluated by FRET-melting, and the corresponding changes in *T*_m_ are shown in Figure 2. The FRET-melting experiments were performed in duplicate and therefore do not allow robust statistical analysis, and the observed values in *T*_m_ provide supportive evidence of the differential effects of DOX and BRACO-19 on AT11-L2 stability.

The *T*_m_ of the AT11-L2 G4 pre-folded in the annealing buffer was found to be 44 °C, which is consistent with the value previously obtained by CD-melting (38.9 °C) [17]. The slight difference in *T*_m_ can be explained by the presence of FAM and TAMRA fluorophores. After adding 1 molar equivalent of DOX and BRACO-19, it was observed that neither showed a significant increase in melting temperature, with a Δ*T*_m_ = 0.7 °C being recorded. However, when 5 molar equivalents were added, differences were observed in the stabilization of AT11-L2 G4, since in the case of DOX, a decrease in *T*_m_ was observed, which may indicate possible aggregation due to the increase in DOX concentration. In contrast, BRACO-19 induced a moderate stabilization effect, resulting in a Δ*T*_m_ = 7.9 °C. These results suggest that BRACO-19 is a promising ligand for stabilizing AT11-L2 G4, which is in agreement with previous findings. However, CD-melting found that BRACO-19 stabilized the G4 structure at over 30 °C when 5 molar equivalents were added [17]. This discrepancy emphasizes the hypothesis that the fluorophores of the labeled sequence can influence the results by interfering with the stacking of the ligand with the G-tetrad [23].

**Figure 3:**
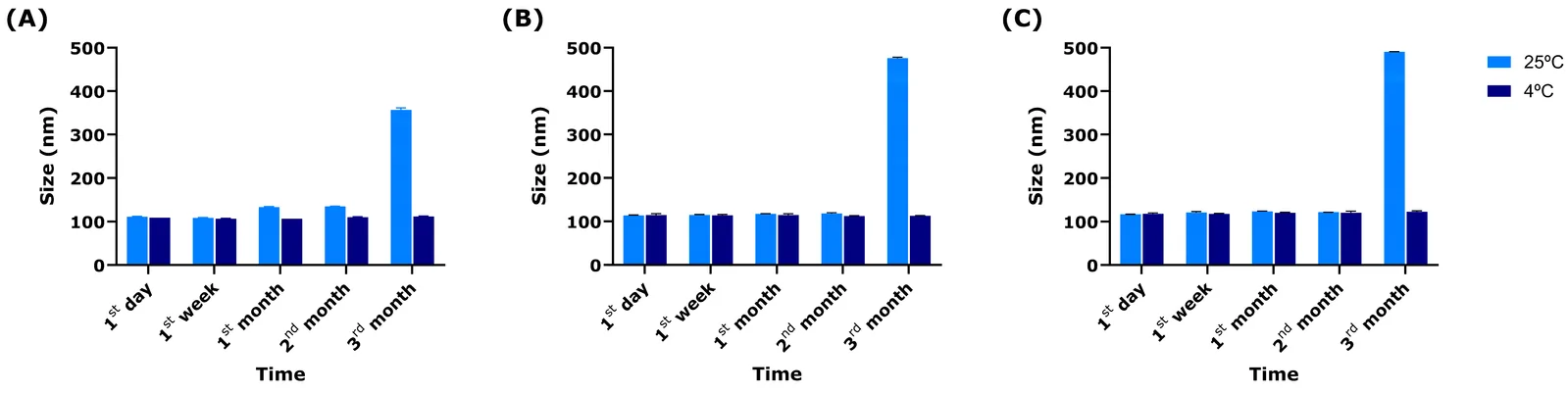
Size of non-functionalized (**A**) blank liposomes, (**B**) DOX liposomes, (**C**) and BRACO-19 liposomes at 25 °C and 4 °C over 3 months (plotted using GraphPad Prism 8.0.1). The results represent mean ± SD of three independent experiments (n = 3).

Although DOX does not stabilize AT11-L2 G4, it is an effective anti-tumor drug used in chemotherapy. BRACO-19, on the other hand, has been studied as a telomerase inhibitor, since it stabilized G4 formed at telomeres, and our results indicate that it also stabilizes AT11-L2 G4. Therefore, in this study, AT11-L2 was conjugated to liposomes containing encapsulated DOX and BRACO-19. Liposomes composed of phosphatidylcholine and cholesterol were prepared and then functionalized with AT11-L2 containing a cholesteryl tail. After producing and functionalizing the liposomes, we proceeded to their characterization and evaluated their stability at different temperatures over time. Size is an important parameter to consider when producing liposomes, as they should ideally have diameters between 50 and 200 nm to avoid rapid elimination from the bloodstream [18]. In this way, we evaluated the size of the blank liposomes, DOX liposomes, and BRACO-19 liposomes over 3 months (Figure 3).

The size of the blank liposomes was evaluated to see if the encapsulation of DOX and BRACO-19 influenced this parameter. Figure 3A shows that the average size of the blank liposomes was around 110 nm in the first week; however, after one month, we observed an increase in the size of the blank liposomes stored at 25 °C, and it continued to increase over time, so after 3 months, a size of 357 nm was recorded, exceeding the ideal size for lipid nanoparticles. The DOX liposomes (Figure 3B) were similar in size (114 nm) to the blank liposomes, indicating that the encapsulation of DOX did not alter the size of the liposomes. However, after three months, once again, a marked increase (476 nm) in the size of the liposomes stored at 25 °C was observed. The results in Figure 3C show that the encapsulation of BRACO-19 did not influence the size of the liposomes either, and, similarly to the liposomes loaded with DOX, a marked increase in liposome size was observed 3 months after their preparation. Therefore, it can be concluded that the liposomes have greater stability when stored at 4 °C, which is in line with what has been observed in previous studies [24].

Another parameter used to assess liposome stability is the polydispersity index (PDI), which reflects the degree of heterogeneity in particle size. For liposomal formulations, a PDI below 0.3 is generally considered indicative of a relatively homogeneous population [4]. The results in Figure S1 show, once again, that liposomes are more stable when stored at 4 °C since we observed an increase in PDI in liposomes stored at 25 °C after 3 months.

To determine whether AT11-L2 functionalization affected the physicochemical properties of the final formulations, the size and PDI of non-functionalized and AT11-L2-functionalized blank, DOX-loaded, and BRACO-19-loaded liposomes were directly compared (Figures S2 and S3). AT11-L2 functionalization increased the particle size to approximately 144 nm, corresponding to an increase of about 36 nm compared with the non-functionalized formulations, consistent with the presence of the aptamer at the liposome surface [25]. Importantly, the AT11-L2-functionalized formulations maintained relatively stable size and PDI values over one week, supporting the short-term physicochemical stability of the final conjugated constructs.

Additionally, since the surface charge of liposomes can be influenced by molecules present at their surface, the zeta potential of non-functionalized and AT11-L2-functionalized liposomes was evaluated (Table S1) to provide further evidence of successful aptamer functionalization. As expected, the zeta potential became more negative after functionalization, consistent with the negatively charged phosphate backbone of AT11-L2 and supporting its presence at the liposome surface. Together, the increase in particle size and the shift toward a more negative zeta potential after AT11-L2 functionalization provide complementary physicochemical evidence consistent with the presence of the aptamer at the liposome surface.

On the other hand, it is essential to select the optimum amount of aptamer to use in the functionalization to avoid the presence of unbound aptamer in the final formulation. For this purpose, a non-denaturing polyacrylamide gel (15%) was made after incubating 1, 2, 3, and 3.3 nmol of aptamer with 1 mg of liposomes. As shown in Figure S4, 3 nmol is the ideal amount of aptamer to use for functionalization, since with 3.3 nmol, bands corresponding to the free aptamer were detected in both DOX- and BRACO-19-loaded liposomes. In addition, we observed an intense band in the wells containing blank liposomes. The presence of these higher molecular weight bands may indicate that the liposomes were degraded, which prevented the aptamer from being incorporated, and consequently, the aptamer sequences containing cholesteryl in the tail formed a nanoaggregate [26]. This result is interesting since the liposome blanks used for functionalization in this test were stored at room temperature. Thus, this result can be correlated with the results obtained previously, indicating that liposomes are not stable when stored at 25 °C.

To assess G4 formation by AT11-L2 on the liposome surface, CD spectra were acquired in the absence and presence of KCl (Figure S5). In the absence of KCl, no clear evidence of a well-defined G4 structure was observed. In contrast, the addition of 100 mM KCl promoted G4 folding, as indicated by a strong positive band around 264 nm, an additional positive contribution around 290 nm, and a negative band near 240 nm. This spectral profile is consistent with the predominantly parallel G4 topology previously reported for AT11-L2 under the same KCl concentration [17]. These results indicate that, after conjugation to the liposome surface, AT11-L2 retains its ability to adopt a G4 structure in a K^+^-rich environment. Maintenance of the G4 structure is particularly relevant because the folded conformation is required for efficient NCL recognition, supporting the structural suitability of liposome-conjugated AT11-L2 as a targeting moiety.

**Figure 4:**
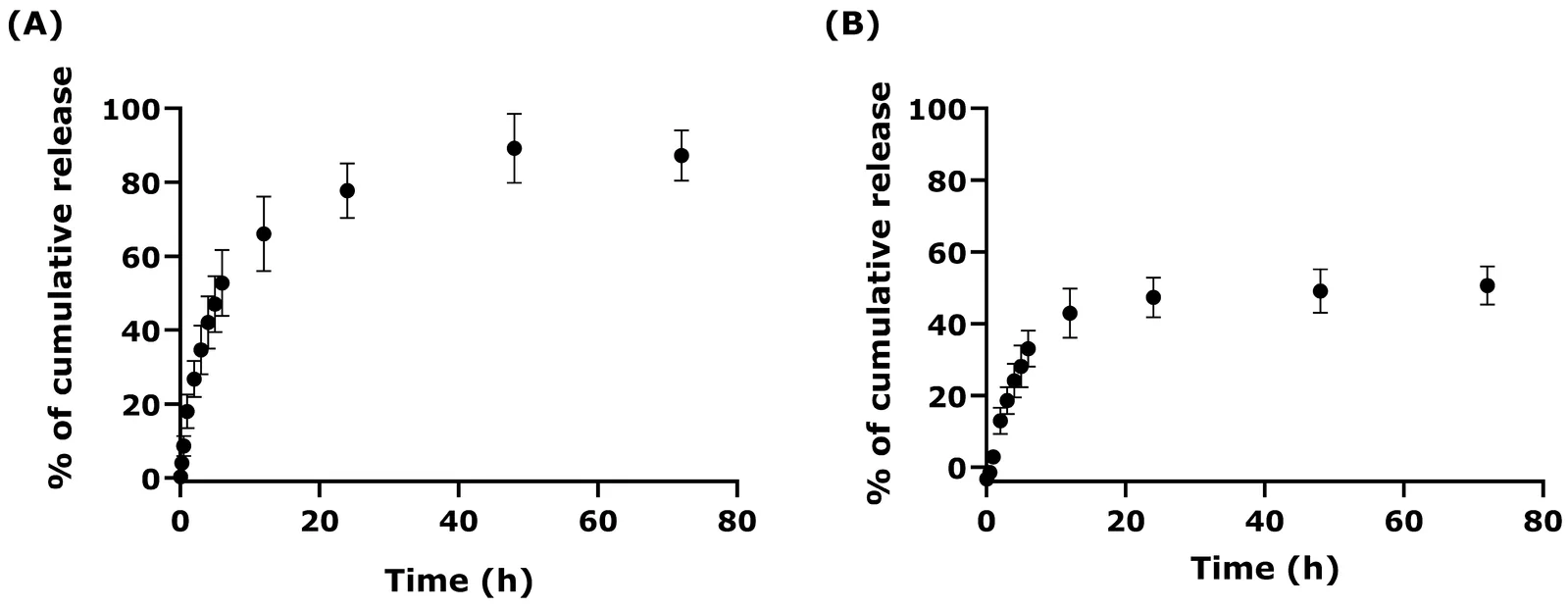
(**A**) Percentage cumulative release of DOX over time. (**B**) Percentage cumulative release of BRACO-19 over time (plotted using GraphPad Prism 8.0.1.). The results represent the mean ± SD of three independent experiments (n = 3).

The encapsulation efficiency (EE) and the percentage of release were also studied. EE was determined by measuring the amount of free DOX and BRACO-19 after separation from the liposomes using centrifugal concentrators. The EE was 90% for DOX liposomes and 88% for BRACO-19 liposomes, suggesting that the majority of the compounds were successfully encapsulated. These results suggest that the produced liposomes can carry large quantities of drugs. The release of DOX and BRACO-19 was monitored over time and is shown in Figure 4. In the case of DOX liposomes, a rapid release was observed in the first 6 h, followed by stabilization until 72 h, when a cumulative release percentage of 89% was achieved, showing that most of the encapsulated DOX was released. In the case of BRACO-19 liposomes, a similar rapid release in the first 6 h was observed, followed by stabilization until 72 h, when 52% of BRACO-19 was released. Notably, BRACO-19 liposomes showed a lower release capacity when compared to DOX liposomes. This difference can be explained by the fact that different concentrations of compounds were encapsulated, suggesting that the amount of compound encapsulated influences its release. However, it was expected that the liposomes containing the hydrophobic compound (BRACO-19) would have a greater release, since hydrophilic compounds are generally associated with reduced permeability across lipid membranes [27].

Then, to assess the effect of DOX and BRACO-19 on the A549 cells’ viability, we determined the IC_50_ after 48 h of exposure to compounds. The concentration-dependent cell viability curves are shown in Figures S6 and S7, respectively. Figure S5A shows that DOX has an effective cytotoxic effect. In this sense, the IC_50_ value determined for DOX was 0.07 µM, a value observed in a previous study [28]. When compared to DOX, BRACO-19 has a lower cytotoxic effect, with an IC_50_ value of 3.42 µM, a value like that previously observed in the same cells [29].

To compare the cytotoxicity of the free and encapsulated compounds, we also evaluated the effect of DOX- and BRACO-19-loaded liposomes at concentrations corresponding to the IC_50_ values of the respective free compounds. The concentration-dependent cell viability curves are shown in Figures S8 and S9, respectively. At 0.07 µM DOX and 3.42 µM BRACO-19, encapsulation into liposomes resulted in lower cytotoxicity than that observed for the corresponding free compounds, with cell viability values of approximately 69% and 60%, respectively. Nevertheless, DOX-loaded liposomes exhibited an IC_50_ of 0.95 µM, indicating that DOX retained substantial cytotoxic activity after encapsulation. In contrast, BRACO-19-loaded liposomes showed a higher IC_50_ of 5.66 µM. Overall, DOX-loaded liposomes displayed greater antiproliferative activity than BRACO-19-loaded liposomes, consistent with the trend observed for the respective free compounds.

**Figure 5:**
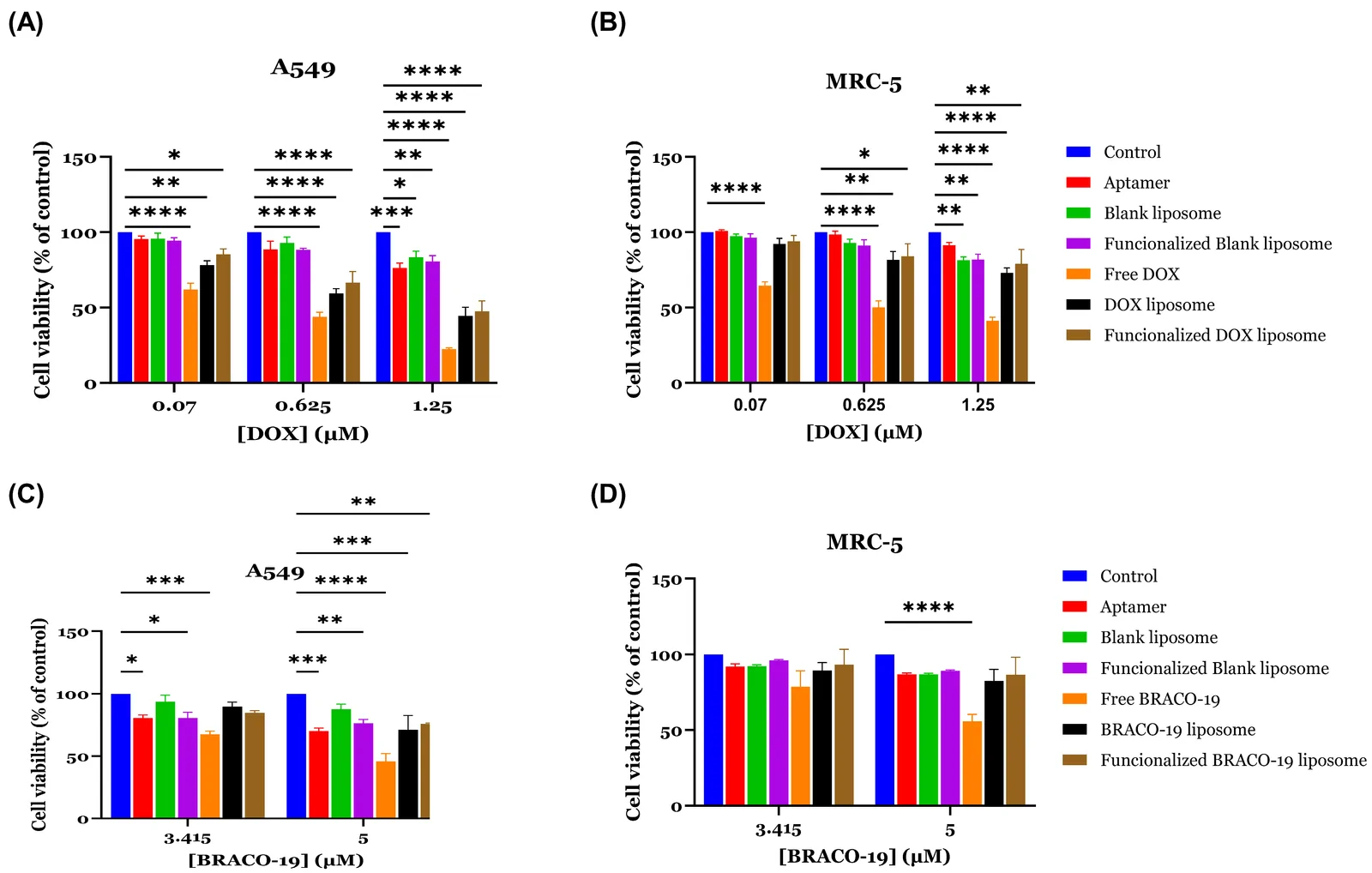
Cell viability of (**A**,**C**) A549 and (**B**,**D**) MRC-5 cells after 48 h of treatment with free AT11-L2, blank liposomes, AT11-L2-functionalized blank liposomes, (**A**,**B**) free DOX or (**C**,**D**) BRACO-19, non-functionalized drug-loaded liposomes, and AT11-L2-functionalized drug-loaded liposomes (plotted using GraphPad Prism 8.0.1). The results are presented as the mean ± SEM of three independent experiments (n = 3), each performed in triplicate. Statistical significance was assessed using two-way ANOVA followed by Dunnett’s multiple comparisons test relative to the corresponding control condition. * *p* < 0.05, ** *p* < 0.01, *** *p* < 0.001, **** *p* < 0.0001.

To evaluate the selectivity of the functionalized liposomes, cell viability was determined in A549 cells (NSCLC) and in non-malignant lung fibroblasts (MRC-5). In addition, to assess the contribution of each component of the nanosystem, six experimental conditions were evaluated for each drug concentration: free AT11-L2, blank liposomes, AT11-L2-functionalized blank liposomes, free drug, non-functionalized drug-loaded liposomes, and AT11-L2-functionalized drug-loaded liposomes. For clarity, DOX-loaded liposomes and BRACO-19-loaded liposomes refer to non-functionalized formulations, whereas AT11-L2-functionalized DOX-loaded liposomes and AT11-L2-functionalized BRACO-19-loaded liposomes refer to the corresponding formulations after aptamer functionalization. The results are presented in Figure 5.

The concentrations of AT11-L2 tested in the DOX experiments (0.07, 0.625, and 1.25 µM) did not markedly affect cell viability in either cell line, with viability remaining above 80%. Similarly, non-functionalized blank liposomes and AT11-L2-functionalized blank liposomes did not exhibit relevant cytotoxic effects in either cell line, supporting the biocompatibility of the lipid formulation and the low intrinsic toxicity of AT11-L2.

When the DOX formulations were compared, both non-functionalized and AT11-L2-functionalized DOX liposomes showed lower cytotoxicity than free DOX, although a substantial reduction in cell viability was observed at the highest concentration tested (1.25 µM). A549 cells generally exhibited lower viability than MRC-5 cells following DOX treatment; however, the relatively modest differences between non-functionalized and AT11-L2-functionalized DOX liposomes indicate that the MTT assay alone does not demonstrate an AT11-L2-mediated enhancement of cytotoxicity. Importantly, the primary role of AT11-L2 in this nanoparticle is not to increase intrinsic cytotoxicity, but to act as an NCL-targeting sequence. Upon adopting its G4 structure, AT11-L2 can recognize NCL and is intended to promote NCL-mediated cellular recognition and internalization. Therefore, the contribution of AT11-L2 should be interpreted mainly in terms of targeted cellular uptake rather than as a direct determinant of cytotoxicity.

For BRACO-19, higher concentrations were required to reach the IC_50_ compared with DOX. Consequently, higher doses of functionalized liposomes, and therefore higher concentrations of the conjugated aptamer, were needed. For this reason, AT11-L2 was evaluated at concentrations of 2.5 and 5 µM. Accordingly, AT11-L2 was evaluated at 2.5 and 5 µM. As shown in Figure 5, AT11-L2 did not show significant cytotoxicity in either cell line. However, in A549 cells, a concentration-dependent decrease in cell viability was observed, indicating that it has an antiproliferative effect at higher concentrations, as described for another AS1411 derivative in a previous study [16]. Once again, non-functionalized blank liposomes and the AT11-L2-functionalized blank liposomes did not affect cell viability. Nevertheless, there was a decrease in cell viability in the functionalized blank liposomes, which can be explained by the presence of the aptamer. Comparing AT11-L2-BRACO-19 liposomes to BRACO-19 liposomes and free BRACO-19, we found that they are less cytotoxic, similarly to what was observed with DOX. In addition, the selectivity for cancer cells is not very pronounced, with only a 10% difference in cell viability at the highest concentration. Overall, DOX-loaded formulations showed greater cytotoxic activity than the corresponding BRACO-19-loaded formulations. However, the relatively modest differences between non-functionalized and AT11-L2-functionalized liposomes indicate that the MTT assay alone does not provide sufficient evidence of aptamer-mediated selectivity. Therefore, the targeting contribution of AT11-L2 was further investigated through cellular uptake studies and NCL-blocking experiments. The cellular uptake of the functionalized liposomes was subsequently assessed by confocal microscopy in A549 and MRC-5 cells. MRC-5 cells were selected as a widely used non-malignant lung-cell model for comparison with A549 cells.

**Figure 6:**
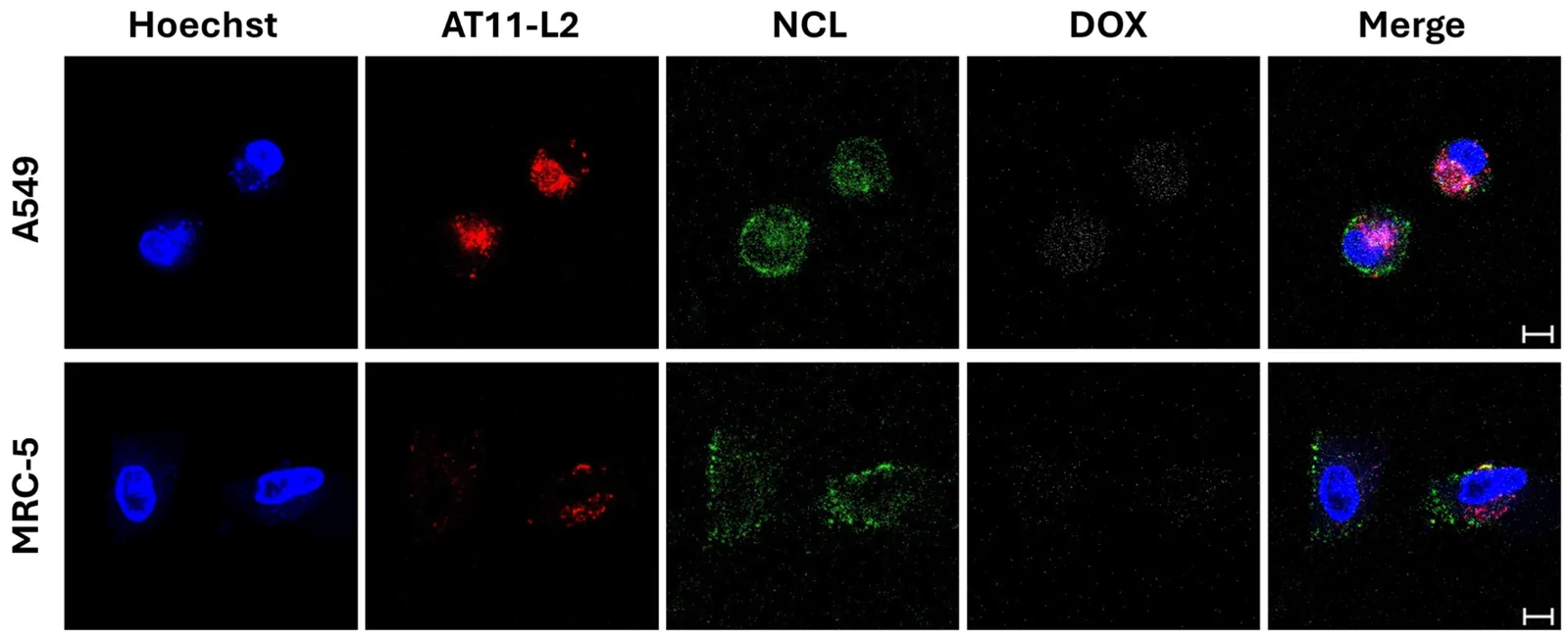
Confocal microscopy fluorescence images of A549 and MRC-5 cells after 2 h of incubation with AT11-L2-DOX liposomes (processed using Zeiss Zen 3.8 software). Nuclei are stained in blue (Hoechst), AT11-L2 in red (Cy5), NCL in green (Alexa Fluor 488®) and DOX in grey. Scale bar: 10 µm.

In the case of AT11-L2-DOX liposomes (Figure 6), the fluorescence of AT11-L2, located on the surface of the liposomes, was more intense in A549 cells. To confirm this result, the fluorescence intensity of AT11-L2 was quantified per cell, revealing approximately a two-fold increase in A549 cells compared to MRC-5 cells (Figure S10), which is consistent with enhanced internalization of functionalized DOX liposomes by NSCLC cells. Previous studies have shown that NCL is overexpressed on the surface of NSCLC cells [30]. Accordingly, NCL fluorescence was expected to be more intense in A549 cells than in MRC-5 cells. However, this difference was not particularly pronounced. Higher DOX intensity was also detected in A549 cells, suggesting that the drug was released within the cancer cells. Nevertheless, some overlap between NCL (488 nm) and DOX signals was observed due to the long excitation–emission range (470–560 nm) of the drug, which prevents definitive attribution of the signal exclusively to released DOX. However, it is important to note that the merged images indicate that AT11-L2 co-localizes with the NCL, suggesting that AT11-L2-DOX liposomes are internalized by A549 cells via NCL.

**Figure 7:**
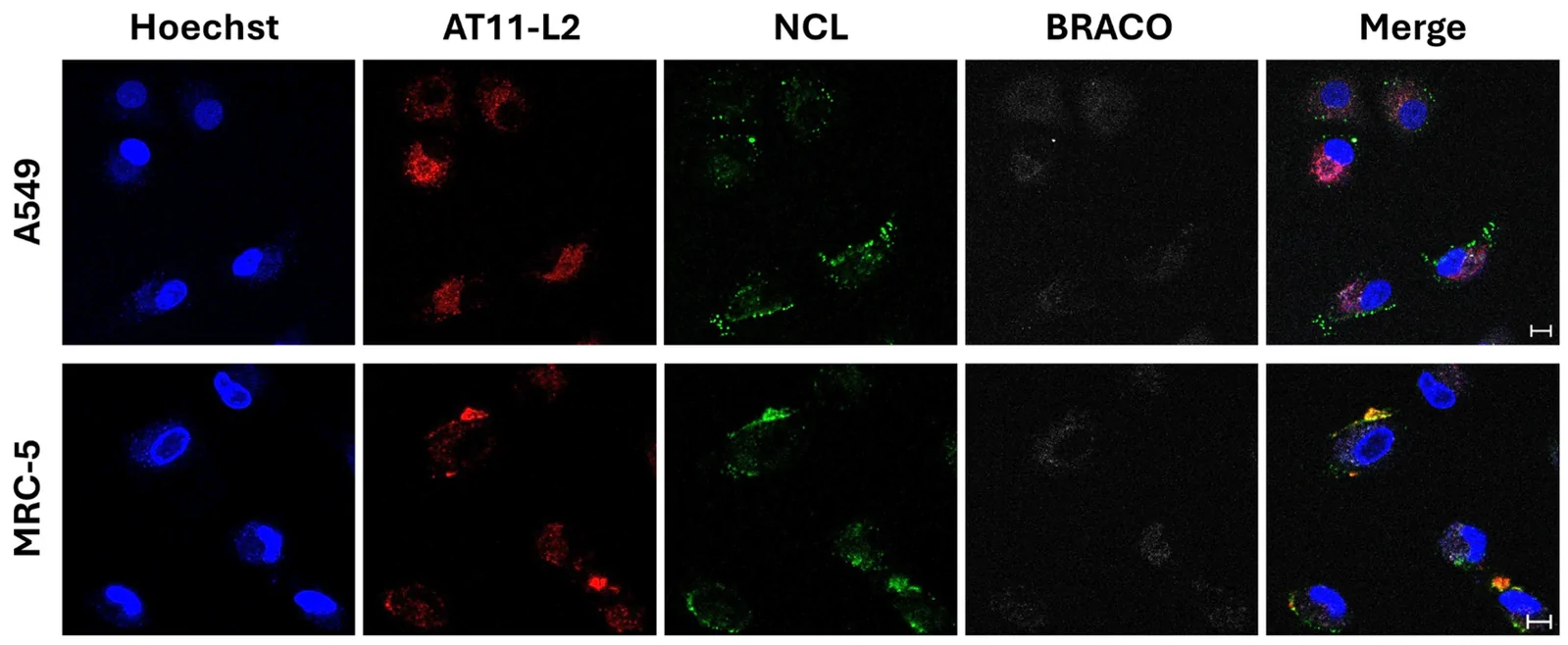
Confocal microscopy fluorescence images of A549 and MRC-5 cells after 2 h of incubation with AT11-L2-BRACO-19 liposome (processed using Zeiss Zen 3.8 software). Nuclei are stained in blue (Hoechst), AT11-L2 in red (Cy5), NCL in green (Alexa Fluor 488®) and BRACO in grey. Scale bar: 10 µm.

Regarding AT11-L2-BRACO-19 liposomes (Figure 7), a stronger aptamer-associated fluorescence signal was observed in A549 cells, consistent with the results obtained for AT11-L2-DOX liposomes, and this was confirmed by per-cell fluorescence quantification (Figure S11). A similar trend was observed for NCL fluorescence intensity. However, substantial fluorescence was also detected in MRC-5 cells. Indeed, quantification of the fluorescence intensity per cell revealed comparable signal levels in A549 and MRC-5 cells (Figure S12), which was unexpected. Nevertheless, orthogonal views from the z-stack analysis revealed distinct NCL localization patterns between the two cell lines, with A549 cells exhibiting a more clearly defined surface-associated NCL signal, whereas MRC-5 cells displayed a more diffuse intracellular distribution of NCL (Figure S13).

**Figure 8:**
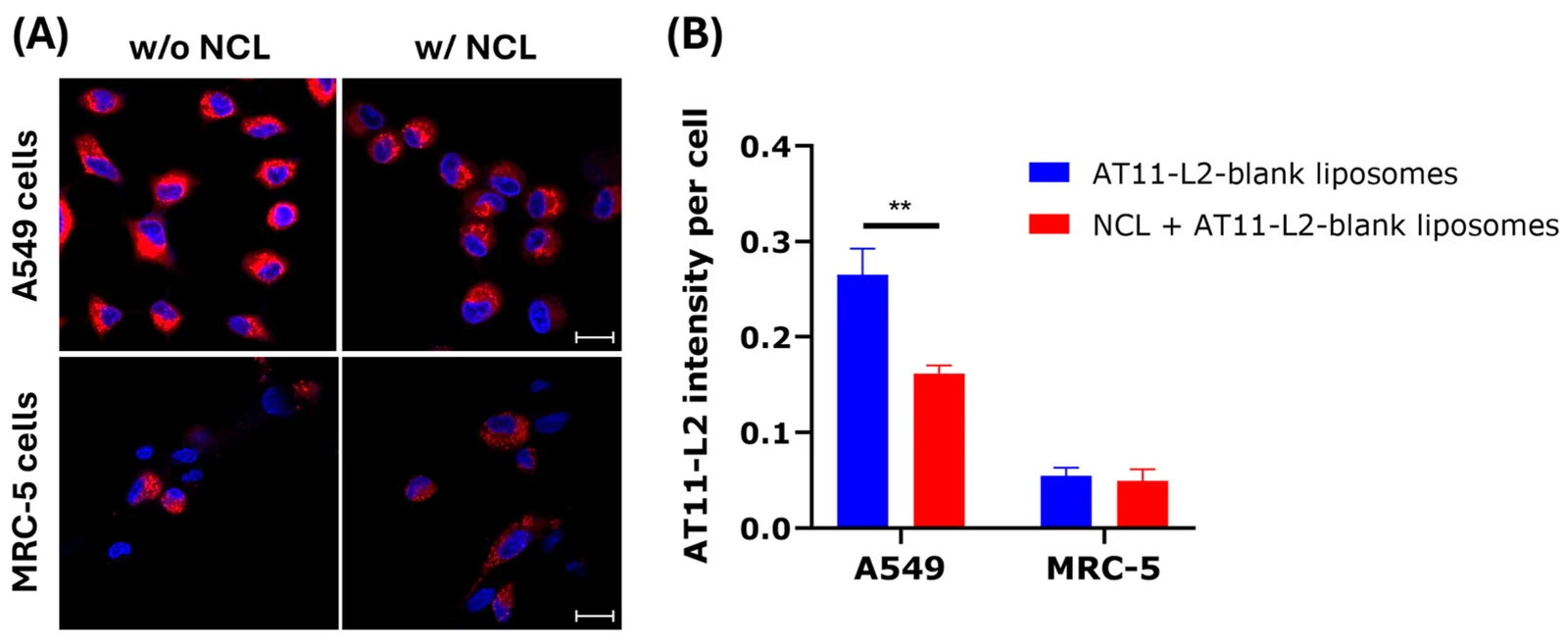
(**A**) Confocal microscopy fluorescence images of A549 and MRC-5 cells without or with 2 h pre-incubation with an anti-NCL antibody, followed by 24 h of incubation with AT11-L2 blank liposomes (processed using Zeiss Zen 3.8 software). Scale bar: 20 µm. (**B**) AT11-L2 intensity per cell in the two cell lines A549 and MRC-5 (plotted using GraphPad Prism 8.0.1). The results represent the mean ± SEM from three independent experiments (n = 3). ** *p* = 0.0098 using the unpaired two-tailed *t*-test.

However, since MRC-5 cells are fibroblasts whereas A549 cells are epithelial adenocarcinoma cells, differences in nanoparticle uptake and intracellular trafficking related to cell lineage cannot be excluded. Therefore, the preferential uptake observed in A549 cells should not be interpreted solely as a consequence of their malignant phenotype. To further investigate the specific contribution of AT11-L2-mediated targeting, NCL-dependent internalization was evaluated using cell-surface NCL blockade. Cells were pre-incubated with an anti-NCL antibody to block cell-surface NCL, followed by incubation with AT11-L2-functionalized blank liposomes. As shown in Figure 8, NCL blockade resulted in a significant reduction in AT11-L2-associated fluorescence intensity in A549 cells, whereas no significant difference was observed in MRC-5 cells. These results provide functional evidence that the uptake of AT11-L2-functionalized liposomes is, at least in part, NCL-dependent in A549 cells. In contrast, under the experimental conditions tested, NCL blockade did not significantly affect AT11-L2-associated liposome uptake in MRC-5 cells.

These findings provide functional evidence that AT11-L2 contributes to the cellular uptake of the functionalized liposomes through NCL-dependent recognition in A549 cells, supporting its specific role as a targeting moiety in the developed nanoparticle.

Finally, and since confocal microscopy did not reveal a clear difference in NCL levels at the cell surface between A549 and MRC-5 cells, we performed Western blot analysis (Figure S14) to further characterize the NCL expression profile in both cell lines, followed by densitometric quantification of the corresponding bands (Figure S15). Western blot analysis revealed several NCL molecular forms with different apparent molecular weights in both cell lines. A549 cells showed a higher abundance of the high-molecular-weight NCL species (~100 kDa). This band may be associated with post-translationally modified forms of NCL, including glycosylated NCL species that have been reported in association with cell-surface NCL in cancer cells [31,32]. In contrast, MRC-5 cells showed a higher abundance of the lower-molecular-weight NCL species (~48 kDa), which may correspond to proteolytic fragments generated by NCL processing or autoproteolysis [33,34]. The additional bands detected at intermediate molecular weights may represent other NCL molecular forms and/or products arising from post-translational or proteolytic processing [32].

Overall, these results demonstrate distinct NCL molecular weight profiles in A549 and MRC-5 cells, with A549 cells showing a greater relative abundance of the ~100 kDa species. Since Western blot analysis was performed using total cellular lysates, these data do not identify which NCL molecular form is specifically present at the plasma membrane or directly involved in AT11-L2 binding. Therefore, no specific NCL molecular-weight species can be assigned as the predominant AT11-L2-binding form based on these data alone. Importantly, AT11-L2 targeting is expected to depend on the accessibility of NCL at the cell surface rather than solely on total cellular NCL abundance. Together with the z-stack analysis and the NCL-blocking experiment, which showed a significant reduction in AT11-L2-associated fluorescence in A549 cells following surface NCL blockade, these findings support an NCL-dependent contribution to the internalization of AT11-L2-functionalized liposomes in A549 cells.

## 3. Materials and Methods

### 3.1. Oligonucleotides and Compounds

The AT11-L2 sequence (5′-TGGTGGTGGTTGTTGTTGGTGGTGGTGGT-3′) and labeled sequences (FAM-AT11-L2-TAMRA, and Cy5-AT11-L2-TEG-cholesteryl) were purchased from Eurogentec (Seraing, Belgium) with HPLC-grade purification. Lyophilized oligonucleotides were resuspended in Milli-Q water (Millipore Milli-Q system; Burlington, MA, USA) and stored at −20 °C until being used. The concentration of the oligonucleotide sequences was obtained by measuring the absorbance at 260 nm using a UV–Vis spectrophotometer (Thermo Scientific^TM^ Evolution 220) (Waltham, MA, USA, EUA) and determined using the manufacturer’s molar extinction coefficient (ε). Before performing each assay, the oligonucleotide was annealed in a buffer with 10 mM lithium cacodylate (Sigma-Aldrich, St. Louis, MO, USA) and 100 mM KCl (PanReac, Castellar del Vallès, Spain) by heating at 95 °C for 10 min, followed by cooling on ice for 10 min. Ligand BRACO-19 (*N*, *N*′-(9-(4-(dimethylamino)phenylamino)acridine-3,6-diyl)bis(3-(pyrrolidine-1-yl)propanamide); CAS: 1177798-88-7; purity ≥ 96%) was purchased from Sigma-Aldrich (St. Louis, MO, USA). DOX ((7S,9S)-7-[(2R,4S,5S,6S)-4-amino-5-hydroxy-6-methyloxan-2-yl] oxy-6,9,11-trihydroxy-9-(2-hydroxyacetyl)-4-methoxy-8,10-dihydro-7H-tetracene-5,12-dione; CAS: 25316-40-9; purity > 95%) was acquired from Tokyo Chemical Industry (Tokyo, Japan). Furthermore, BRACO-19 and DOX were used without further purification, and stock solutions were prepared at 10 mM in dimethyl sulfoxide (DMSO; CAS: 67-68-5; ThermoFisher Scientific, Waltham, MA, USA).

### 3.2. Förster Resonance Energy Transfer (FRET)-Melting Experiments

The FAM-AT11-L2-TAMRA tagged sequence and the BRACO-19 and DOX ligands were used to carry out the FRET-melting studies. A 96-well plate was used for this assay, where 20 μL of oligonucleotide at a concentration of 0.25 μM pre-annealed in a buffer containing 10 mM lithium cacodylate and 100 mM KCl, was added to each well. Each experimental condition was performed in duplicate. Subsequently, 5 μL of ligand was added at 0.2 (1 molar equivalent) and 1 μM (5 molar equivalents). The multi-well plate was then placed on a thermocycler (CFX Connect™ Real-Time PCR (Bio-Rad Laboratories, Hercules, CA, USA)), equipped with a FAM filter (λ_ex_ = 495 nm; λ_em_ = 518 nm). The microplate was subjected to temperature variations of 1 °C/min. To determine the melting temperature (*T*_m_), the data obtained were fitted to a Boltzmann distribution using OriginPro2021, and the mid-transition temperature was taken from the normalized curves. Since the FRET-melting experiments were performed in duplicate for each experimental condition, the data were used to assess trends in AT11-L2 thermal stabilization and were not subjected to inferential statistical analysis.

### 3.3. Production of Liposomes

Liposomes containing BRACO-19 and DOX were prepared using the ethanol injection method previously described by Batzri and Korn [35]. To this end, a lipid solution was prepared with 375 μL of cholesterol (CAS: 57-88-5, Sigma-Aldrich, St. Louis, MO, USA) at a concentration of 12.5 mg/mL, 375 μL of phosphatidylcholine (PHOSPHOLIPON ^®^ 90 G, Lipoid Kosmetik™, Steinhausen, Switzerland) at a concentration of 130 mg/mL, and 750 μL of 99.5% ethanol [36]. As DOX is classified as a hydrophilic drug, it was dissolved in 9 mL of Milli-Q water to obtain a final concentration of 100 μM. Then, using a syringe with an attached optical fiber and an NE-300 Just infusion syringe pump (New Era Pump Systems, Farmingdale, NY, USA), 1 mL of the lipid solution was injected into 9 mL of Milli-Q water containing DOX, under gentle agitation, at a flow rate of 33 μL/min. As BRACO-19 is considered a hydrophobic molecule, it was dissolved in the lipid solution to obtain a final concentration of 50 μM, and the rest of the process took place in the same way to produce the liposomes with BRACO-19. The liposomes were stored at room temperature and 4 °C for future assays.

### 3.4. Functionalization of Liposomes with AT11-L2

To prepare liposomes with AT11-L2 on the surface, the method previously described by Nsairat et al. was followed [25]. Thus, various amounts of AT11-L2- TEG-cholesteryl (1, 2, 3, 3.3 nmol) were incubated at room temperature for 20 min with 1 mg of liposomes. AT11-L2-TEG-Cholesteryl anchoring to liposomes is a spontaneous process since the cholesteryl tail is incorporated into the lipid membrane. To determine the appropriate amount of aptamer to use so that it is fully incorporated into the surface of the liposomes, a non-denaturing polyacrylamide gel electrophoresis was carried out. Firstly, a pre-run was carried out at 120 V for 15 min using Tris/Borate/EDTA as a running buffer. A HyperLadder™ 25 bp, spanning 25–500 base pairs (Bioline, London, UK), served as a relative mobility marker. Electrophoresis was then carried out at 150 V for 50 min. The gel was stained with SYBR gold under gentle agitation for 10 min. Finally, the gel was observed using ChemiDOCT MXRS (Bio-Rad Laboratories, Hercules, CA, USA).

### 3.5. Circular Dichroism (CD) Spectroscopy

CD studies were carried out to confirm that AT11-L2 effectively forms a G4 structure on the surface of liposomes in an environment rich in K^+^. The functionalized liposomes were added to a 1 mm path-length quartz cuvette (Hellma, Müllheim, Germany), then the corresponding volume was added for a final concentration of 100 mM KCl. The CD spectra were acquired on a Jasco J-815 spectropolarimeter (Tokyo, Japan) at a wavelength of 200 to 320 nm, with a scanning speed of 100 nm/min, a bandwidth of 1 nm, and an integration time of 1s. Three average accumulations were made.

### 3.6. Liposomes Characterization

#### 3.6.1. Dynamic Light Scattering (DLS)

Liposomes’ size, polydispersity index (PDI), and zeta potential were determined by DLS. To evaluate the stability of the liposomes under different storage conditions, two liposome samples were stored either at room temperature or at 4 °C, with their size and PDI were monitored over a period of 3 months or 1 week, for non-functionalized and AT11-L2 functionalized liposomes, respectively. For DLS measurements, samples (1 mL) were inserted into a disposable plastic cuvette with a 1 cm diameter beam. Measurements were performed using a Zetasizer Nano ZS (Malvern Instruments, Malvern, UK) and data were analyzed with Malvern Zetasizer v7.13 software. In addition, a dip cell was added to the plastic cell to analyze the zeta potential of the liposomes.

#### 3.6.2. Encapsulation Efficiency (EE)

EE was determined by measuring the amount of unencapsulated DOX and BRACO-19. Centrifugal concentrators (ThermoFisher Scientific^TM^, MA, USA; MWCO 2 kDa) separated the compounds not encapsulated in the liposomes. Next, the absorbance of the solutions obtained after centrifugation was measured by spectrophotometry in a UV–Vis spectrophotometer (Thermo Scientific™ Evolution 220) at a wavelength of 480 or 362 nm, corresponding to the maximum absorption of DOX and BRACO-19, respectively. To determine the concentrations of the non-encapsulated compounds, standard calibration curves with different concentrations of BRACO-19 and DOX were used. Finally, the loading efficiency of the liposomes was determined using Equation (1).

(1) $$EE\%=\frac{Amount\;of\;compound\;added-Amount\;of\;free\;compound}{Amount\;of\;compound\;added}\times 100\;$$

#### 3.6.3. Compound Release

The release profile of the compounds was determined by performing a dialysis method, and subsequently, the number of compounds released over time was quantified. Briefly, 100 μL of liposomes were inserted into a dialysis device, Slide-a-Lyzer^TM^ (ThermoFisher Scientific^TM^, MA, USA; MWCO of 3.5 kDa). The device was then inserted into a 1.5 mL Eppendorf containing 1 mL of phosphate-buffered saline (PBS). The Eppendorf was placed under constant agitation in a PTR-35 Multi-Rotator (Grant Instruments Cambridge, Royston, UK) at room temperature. Samples were then collected at different times (0 min, 5 min, 15 min, 30 min, 1 h, 2 h, 3 h, 4 h, 5 h, 6 h, 12 h, 24 h, and 72 h), and the volume removed was always replaced with PBS. To quantify the compounds released, the fluorescence of the samples was determined using a Horiba FluoroMax4 fluorometer (Tokyo, Japan) with e λ_ex_ = 480 nm and λ_em_ = 495 nm for DOX and λ_ex_ = 362 nm and λ_em_ = 377 nm for BRACO-19. A previously calculated calibration curve was used to determine the percentage of cumulative release.

### 3.7. Cell Studies

#### 3.7.1. Cell Culture

Human alveolar basal epithelial adenocarcinoma cells (A549) and human fetal lung fibroblast cells (MRC-5) were purchased from the American Type Culture Collection (ATCC) and cultured in 75 cm^2^ T-flasks at 37 °C in a humidified atmosphere containing 5% CO_2_. The A549 cell line was cultured in Ham’s F-12 medium (Sigma-Aldrich, MO, USA) supplemented with 10% fetal bovine serum (FBS) and 1% streptomycin–penicillin (SP) antibiotic. On the other hand, the MRC-5 cell line was grown in EMEM medium (Gibco, Thermo Scientific^TM^, MA, USA), supplemented with 1% Non-Essential Amino Acid Solution (Sigma-Aldrich, MO, USA), 10% FBS, and 1% SP antibiotic.

#### 3.7.2. Cell Viability Assay

The IC_50_ values of the free compounds and the corresponding non-functionalized drug-loaded liposomes were determined using the MTT assay. In addition, the biological effects of the final AT11-L2-functionalized formulations were evaluated in A549 and MRC-5 cells. For each drug concentration, six experimental conditions were tested: free AT11-L2, blank liposomes, AT11-L2-functionalized blank liposomes, free drug, non-functionalized drug-loaded liposomes, and AT11-L2-functionalized drug-loaded liposomes. For DOX, the non-functionalized and functionalized formulations are referred to as DOX-loaded liposomes and AT11-L2-functionalized DOX-loaded liposomes, respectively. The corresponding BRACO-19 formulations are referred to as BRACO-19-loaded liposomes and AT11-L2-functionalized BRACO-19-loaded liposomes, respectively.

For this assay, A549 and MRC-5 cells were seeded in 96-well plates at a density of 4 × 10^4^ cells/mL with their respective culture medium. After 24 h of adhesion, the medium was removed, and the samples were dissolved in the appropriate culture medium. After 48 h, the medium was removed, and 100 μL of MTT solution (CAS: 298-93-1; ThermoFisher Scientific^TM^, MA, USA) at 1 mg/mL was added, and the cells were incubated for 2 h at 37 °C. The crystals resulting from the transformation of MTT into formazan were dissolved in 100 μL of DMSO, and absorbance was determined in a plate reader with a Bio-Rad xMark spectrophotometer (Bio-Rad Laboratories, USA) at 570 nm. Each assay was carried out in triplicate in three independent experiments. The data were processed in GraphPad Prism 8.0.1.

#### 3.7.3. Confocal Fluorescence Microscopy

A549 and MRC-5 cells were seeded in treated μ-slide 8-well (IBIDI, Germany) at a density of 5 × 10^4^ cells/mL and incubated in a humidified atmosphere with 5% CO_2_ at 37 °C. After 48 h, the following stimuli were applied for 2 h at 37 °C: free DOX, DOX liposome, AT11-L2-DOX liposome, functionalized blank liposome, and AT11-L2 aptamer labeled with 5′-Cy5. To stain NCL, the cells were incubated with a primary polyclonal anti-NCL antibody (PA3-16875, Invitrogen, Waltham, MA, USA) at a dilution of 1:100 for 2 h at 37 °C. Subsequently, the secondary antibody (Alexa Fluor 488^®^, A32731, Invitrogen^TM^, ThermoFisher Scientific, MA, USA) was added at a dilution of 1:1000, and the cells were incubated again for 1 h. Finally, the cells were labeled with 2 μM of Hoechst 33342^®^ (h3570, Invitrogen^TM^, ThermoFisher Scientific, MA, USA) for 15 min. The same assay was repeated with other conditions: free BRACO-19, BRACO-19 liposome, AT11-L2-BRACO-19 liposome, functionalized blank liposome, and AT11-L2 aptamer labeled with 5′-Cy5. In addition to the different conditions, the secondary antibody Alexa Fluor 546^®^ (A-11035, Invitrogen, USA) was used. Between each step, the cells were washed with PBS three times. The cells were observed on a Zeiss LSM 710 laser scanning confocal microscope (Zeiss, Germany) coupled to a plan-apochromatic and a 63×/DIC M27 objective. After acquisition, the fluorescence intensity per cell was quantified for all conditions using Zeiss Zen 3.8 software.

To assess NCL-dependent internalization, cells were pre-incubated with an anti-NCL antibody (1:100) to saturate the cell’s surface protein for 2 h, followed by a 24 h incubation with AT11-L2-functionalized liposomes. Wells without antibody treatment were included to observe normal liposomes’ internalization. After incubation, cells were stained with 2 μM Hoechst for 15 min and imaged using a Zeiss AxioObserver LSM 710 microscope (Zeiss, Oberkochen, Germany), with 405 nm and 633 nm laser excitation for Hoechst 33342 and Cy5 from AT11-L2, respectively.

#### 3.7.4. Western Blot

To extract the proteins, pellets containing the cells were homogenized in a cell lysis buffer consisting of 600 μL of Pierce^®^ RIPA buffer (25 mM Tris-HCl, pH 7.6, 150 mM NaCl, 1% NP-40, 1% sodium deoxycholate, and 0.1% SDS) (ThermoFisher Scientific, MA, USA) supplemented with 60 μL of complete EDTA-free protease inhibitor cocktail (ROCHE) (ThermoFisher Scientific^TM^, MA, USA) and 6 μL of phenylmethylsulfonyl fluoride (PMSF) (ThermoFisher Scientific^TM^, MA, USA). The extract resulting from the lysis was incubated for 20 min on ice and centrifuged at 4 °C with 14000 RCF. Total protein was quantified using the Pierce^TM^ BCA Protein Assay Kit (ThermoFisher Scientific^TM^, MA, USA). After quantification, 30 µg of total protein from each cell extract was denatured at 100 °C for 5 min in a reducing buffer containing β-mercaptoethanol (Gibco, Thermo Scientific^TM,^ MA, USA). Next, polyacrylamide gel electrophoresis with 10% sodium dodecyl sulfate (SDS-PAGE) was carried out at 90 V for 1 h to fractionate the proteins. The proteins were then transferred to a blotting membrane with a polyvinylidene difluoride filter (PVDF, Amersham^TM^ Hybond^®^ P 0.45 µm PVDF, GE Healthcare) and blocked with a 5% BSA solution prepared in TBS-T (200 mM Tris-HCl, pH 7.4; 1.37 M NaCl; 0.1% Tween 20) for 1 h. This was followed by incubation of the membrane with anti-NCL polyclonal primary antibody (PA3-16875, Invitrogen^TM^, ThermoFisher Scientific, MA, USA) at a dilution of 1:2000 in 0.5% BSA, overnight at 4 °C. The following day, the membrane was incubated with HRP-labeled anti-rabbit IgG secondary antibody (1:5000, Invitrogen^TM^, ThermoFisher Scientific, MA, USA) for 1 h at room temperature. To visualize the NCL bands, the membrane was incubated with ECL substrate (WesternBright™ Sirius, K-12043-D10, Advansta, San Jose, CA, USA) for 2 min, protected from light, and then washed with TBS-T. Subsequently, without performing a stripping step, the membrane was subjected to a second round of probing. For that, it was incubated with anti-β-actin primary antibody (MA1-140, Invitrogen^TM^, ThermoFisher Scientific, MA, USA) for 2 h (1:2000). Subsequently, it was also incubated with HRP-labeled anti-mouse IgG secondary antibody (1:5000, Invitrogen, Waltham, MA, USA) for 1 h. To visualize the β-actin band, the membrane was again incubated with an ECL substrate. The images were acquired on the ChemiDoc^TM^ XRS system (Bio-Rad Laboratories, Hercules, CA, USA) and analyzed with Image Lab software version 5.1 (Bio-Rad Laboratories, Hercules, CA, USA).

#### 3.7.5. Statistical Analysis

Data are presented as the mean ± standard deviation (SD) or as the mean ± standard error of the mean (SEM) as depicted in each figure legend. Statistical analyses were performed in GraphPad Prism (version 8.0.1) with α = 0.05. Normality was assessed by the Shapiro–Wilk test. MTT assay was performed under three independent assays (n = 3) in triplicate and analyzed with a two-way ANOVA test with Dunnett’s multiple comparison test. Confocal studies fluorescence was quantified using at least three independent images and analyzed with an unpaired *t*-test was used in the fluorescence intensity results. The FRET-melting experiments consisted of one independent experiment with each experimental condition measured in duplicate and were therefore not subjected to inferential statistical analysis. CD measurements were also obtained from one independent experiment and were interpreted qualitatively. All the remaining assays were performed in three independent assays (n = 3).

## 4. Conclusions

Liposomes functionalized with AT11-L2 were developed to selectively deliver drugs to cancer cells via NCL.

FRET-melting assays showed that BRACO-19 stabilized the AT11-L2 G4 structure, with a Δ*T*_m_ of 7.9 °C at 5 molar equivalents, whereas DOX had no significant stabilizing effect. DOX- and BRACO-19-loaded liposomes were subsequently prepared by ethanol injection and functionalized with AT11-L2. Drug encapsulation did not markedly affect particle size, which remained close to 110 nm, while functionalization increased the size up to ~36 nm and reduced the surface charge, consistent with aptamer conjugation. The nanoparticles showed stability at 4 °C, maintaining suitable physicochemical properties for up to 3 months. CD analysis confirmed that surface-conjugated AT11-L2 retained its ability to fold into a parallel G4 structure in the presence of K^+^. Both nanoparticles displayed high encapsulation efficiency, although DOX-loaded liposomes showed a higher release profile than BRACO-19-loaded liposomes. In vitro, AT11-L2- DOX liposomes exhibited greater selectivity toward A549 cancer cells and reduced cell viability, whereas BRACO-19-liposomes showed lower selectivity and cytotoxicity. Additionally, NCL-blocking experiments supported an NCL-dependent contribution to the internalization of AT11-L2-functionalized liposomes in A549 cells, providing functional evidence for the targeting role of AT11-L2.

Overall, these findings support AT11-L2-functionalized liposomes as a promising NCL-targeted platform for the delivery of anticancer drugs to cells.

## Data Availability

The data supporting the findings of this study are available within the article and its Supplementary Materials. Additional data is available from the corresponding author upon a reasonable request.

## Supplementary Materials

The following supporting information can be downloaded at: https://www.mdpi.com/article/10.3390/molecules31173134/s1. Table S1: Zeta potential for non-functionalized liposomes and functionalized liposomes. Figure S1: PDI of (A) blank liposomes, (B) DOX liposomes, (C) BRACO-19 liposomes at 25 °C and 4 °C over 3 months. Figure S2: Size of non-functionalized and AT11-L2-functionalized (A) blank liposomes, (B) DOX liposomes, and (C) BRACO-19 liposomes at 25 °C over 1 week. Figure S3: PDI of non-functionalized and AT11-L2-functionalized (A) blank liposomes, (B) DOX liposomes, and (C) BRACO-19 liposomes at 25 °C over 1 week. Figure S4: Non-denaturing gel electrophoresis of functionalized liposomes. Figure S5: CD spectra of functionalized liposomes in the presence and absence of KCl. Figure S6: (A) Cell viability after 48 h of incubation with DOX concentrations of 3.13 × 10^−4^ to 5 μM; (B) concentration-dependent curve of cell viability. Figure S7: Cell viability after 48 h of incubation with BRACO-19 concentrations of 1 × 10^−2^ to 100 μM; (B) concentration-dependent curve of cell viability. Figure S8: (A) Cell viability after 48 h of incubation with DOX concentrations in liposomes of 1 × 10^−3^ to 2.5 μM; (B) concentration-dependent cell viability curve of DOX liposomes. Figure S9: (A) Cell viability after 48 h of incubation with BRACO-19 concentrations in liposomes of 1 × 10^−1^ to 25 μM; (B) concentration-dependent cell viability curve of BRACO-19 liposomes. Figure S10: AT11-L2 intensity per cell in the two cell lines A549 and MRC-5 after 2 h of incubation with functionalized DOX liposome. Figure S11: AT11-L2 intensity per cell in the two cell lines A549 and MRC-5 after 2 h of incubation with functionalized BRACO-19 liposome. Figure S12. NCL total intensity per cell in the two cell lines A549 and MRC-5. Figure S13: Representative z-stack data showing NCL expression in (A) A549 cells and (B) MRC-5 cells. Figure S14: (A) NCL and (B) β-actin expression analyzed by Western blot. Figure S15: NCL quantification in the 48, 63, 75, and 100 kDa bands and total NCL expression.
