# Supplementary material for: Aptamer-Functionalized Liposomes for Targeted Delivery of Anticancer Drugs in Lung Cancer
Source: Molecules. 2026 Sep 7;31(17):3134. doi: 10.3390/molecules31173134 (PMC13567002; doi:10.3390/molecules31173134)
Supplement: Supplementary file 1 [file molecules-31-03134-s001.zip › molecules-4522870-supplementary.pdf]

Supplementary Materials

Aptamer-Functionalized Liposomes for Targeted Delivery of Anticancer Drugs in Lung Cancer

Daniela Leitão[1], David Moreira[1], Jéssica Lopes-Nunes[1], Joana Figueiredo[1], Carla Cruz[1*]

[1] RISE-Health UBI, Department of Chemistry, Faculty of Sciences, University of Beira Interior, Rua Marquês d'Ávila e Bolama, Covilhã, 6201-001, Portugal.

* Corresponding author email: carlacruz@fcsaude.ubi.pt

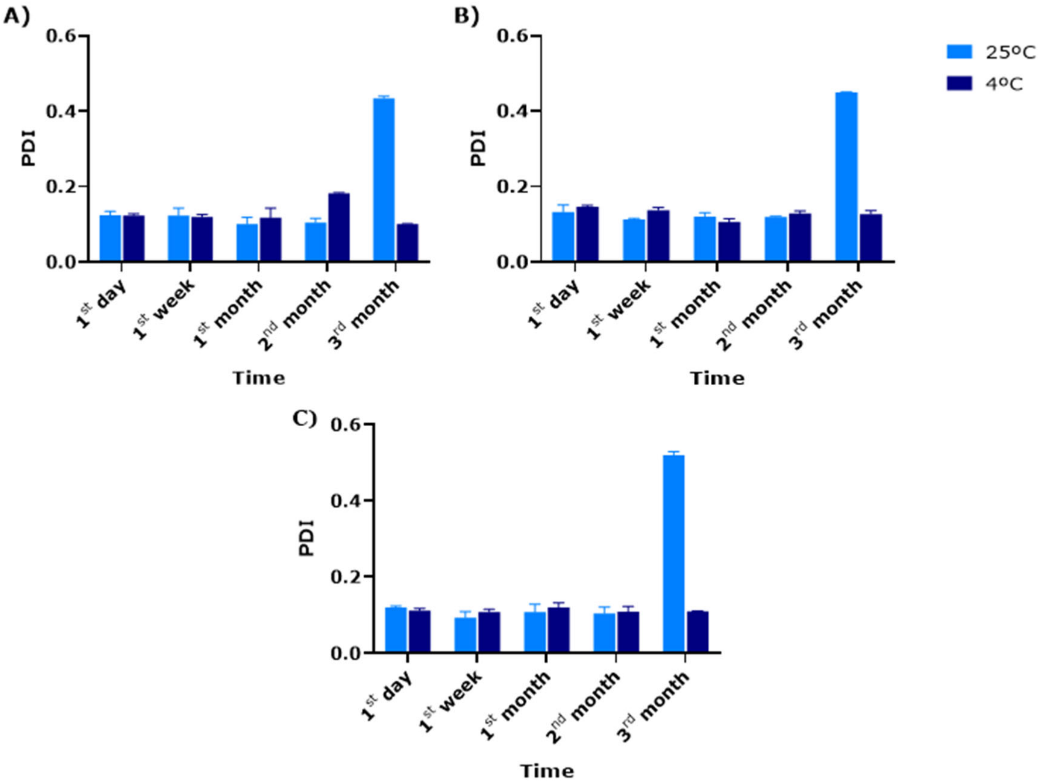

Figure S1. PDI of non-functionalized (□) Blank liposomes (B) DOX liposomes (C) BR□CO-19 liposomes at 25°C and 4°C over 3 months (plotted using GraphPad Prism 8.0.1.).The results represent mean ± SD of at three independent experiments (n=3).

Table S1. Zeta potential of non-functionalized liposomes and functionalized liposomes.

	**Zeta potential**
**Non-functionalized liposomes**	-14.87±0.03
**Liposomes functionalized**	-28.8±0.89

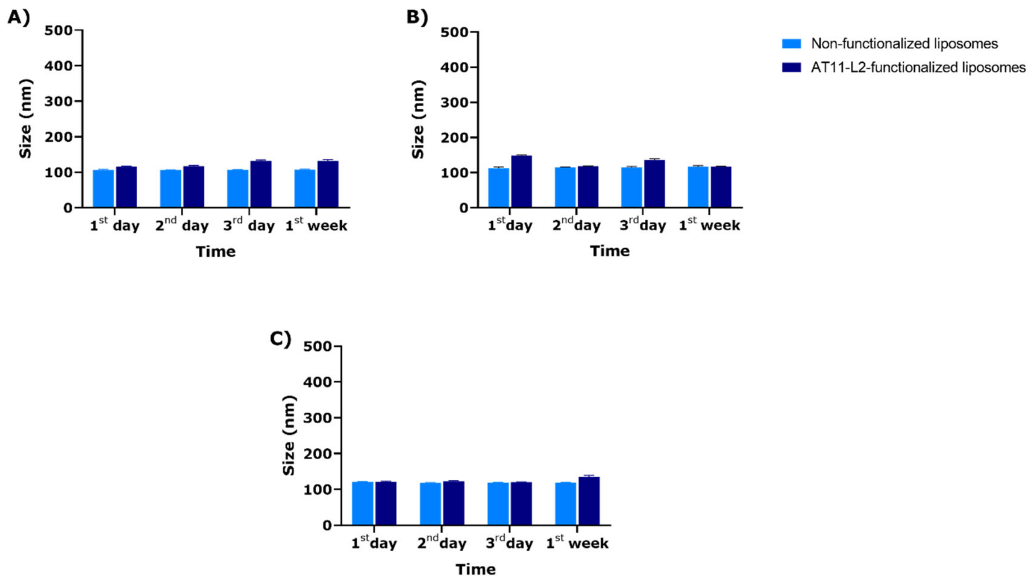

Figure S2. Size of non-functionalized and T11-L2-functionalized () blank liposomes, (B) DOX liposomes, and (C) BRCO-19 liposomes at 25°C over 1 week (plotted using GraphPad Prism 8.0.1.).The results represent mean ± SD of at three independent experiments (n=3).

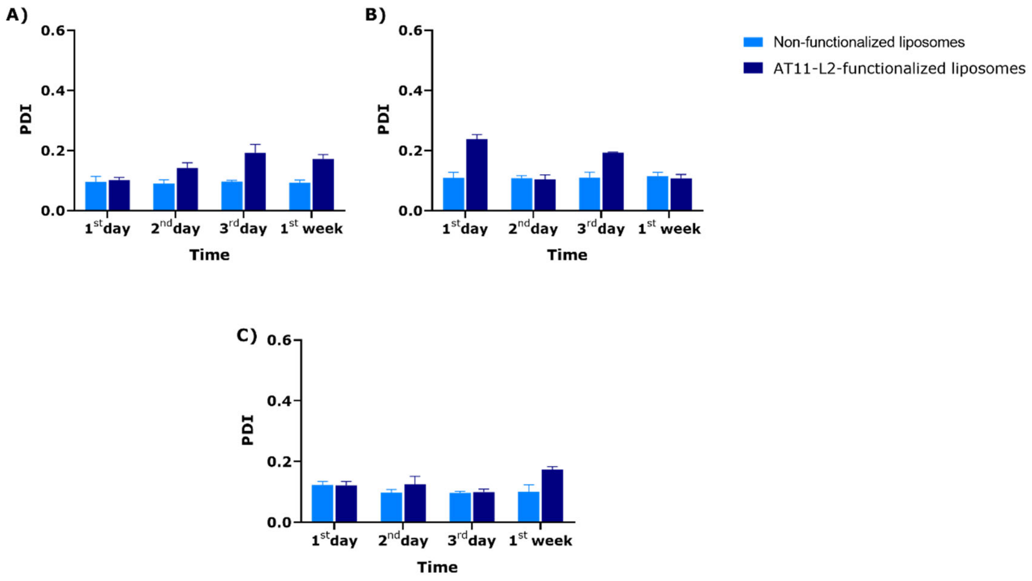

Figure S3. PDI of non-functionalized and T11-L2-functionalized () blank liposomes, (B) DOX liposomes, and (C) BRCO-19 liposomes at 25°C over 1 week (plotted using GraphPad Prism 8.0.1.).The results represent mean ± SD of at three independent experiments (n=3).

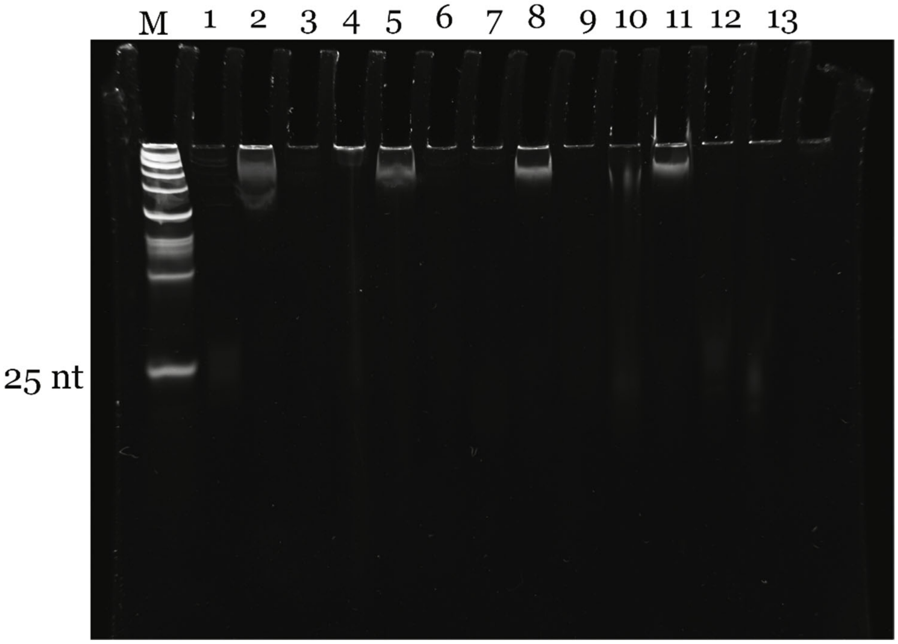

Figure S4. Non-denaturing gel electrophoresis of T11-L2-functionalized liposomes (Lanes: M- marker; 1- T11-L2 in water; 2- blank liposomes with 1 nmol of T11-L2; 3- DOX liposomes with 1 nmol of T11-L2; 4- BRCO-19 liposomes with 1 nmol of T11-L2; 5- blank liposomes with 2 nmol of T11-L2; 6- DOX liposomes with 2 nmol of T11-L2; 7- BRCO-19 liposomes with 2 nmol of T11-L2; 8- blank liposomes with 3 nmol of T11-L2; 9- DOX liposomes with 3 nmol of T11-L2; 10- BRCO-19 liposomes with 3 nmol of T11-L2; 11- blank liposomes with 3.3 nmol of T11-L2; 12- DOX liposomes with 3.3 nmol of T11-L2; 13- BRCO-19 liposomes with 3.3 nmol of T11-L2.

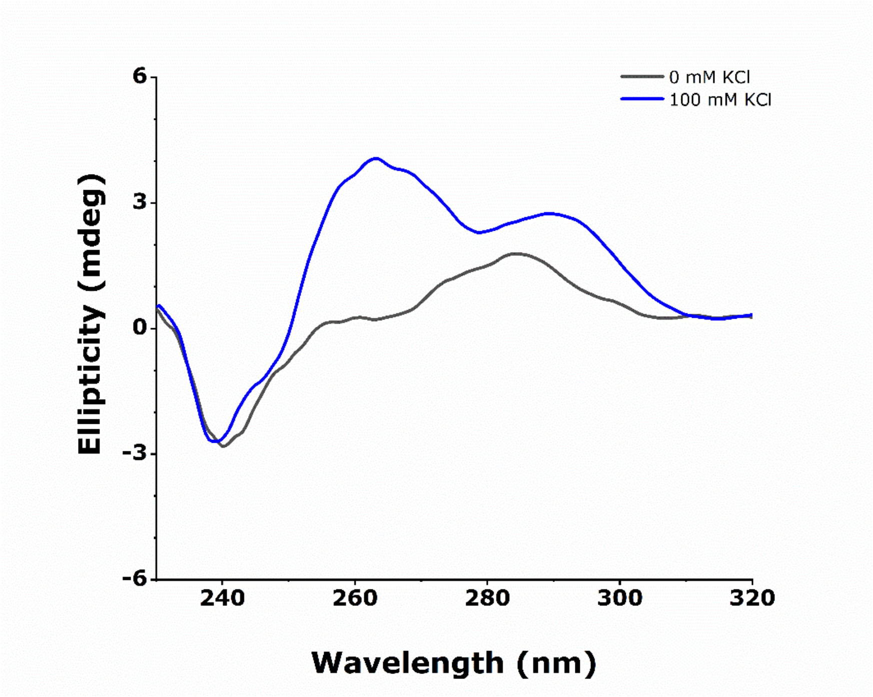

Figure S5. CD spectra of T11-L2-functionalized liposomes in the presence and absence of KCl (plotted with OriginPro2021). The spectra represents one indepent assay (n=1) acquired over 3 accumulations.

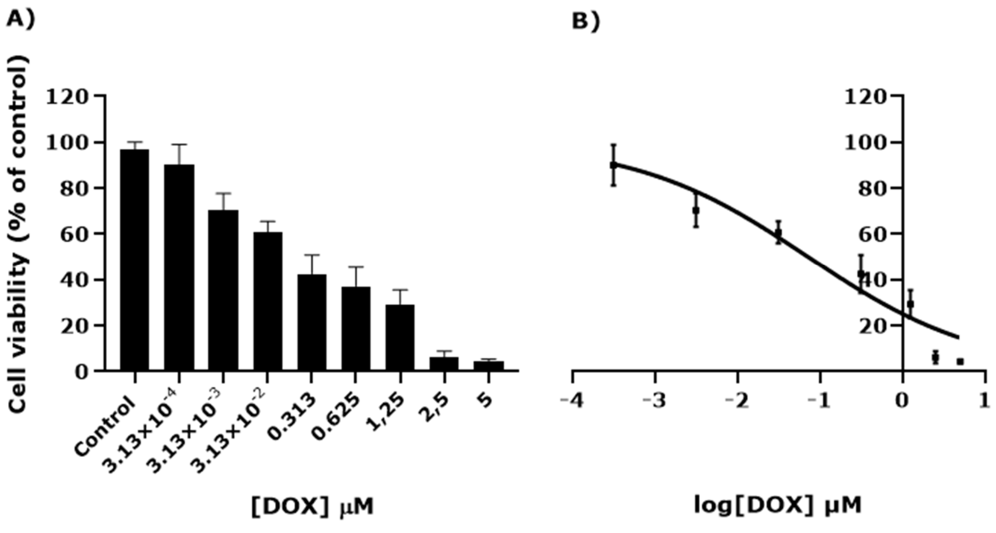

Figure S6. () Cell viability after 48 h of incubation with DOX concentrations of 3.13 × 10^{-4} to 5 μM; (B) Concentration-dependent curve of cell viability (plotted using GraphPad Prism 8.0.1.).The results represent mean ± SD of at three independent experiments (n=3).

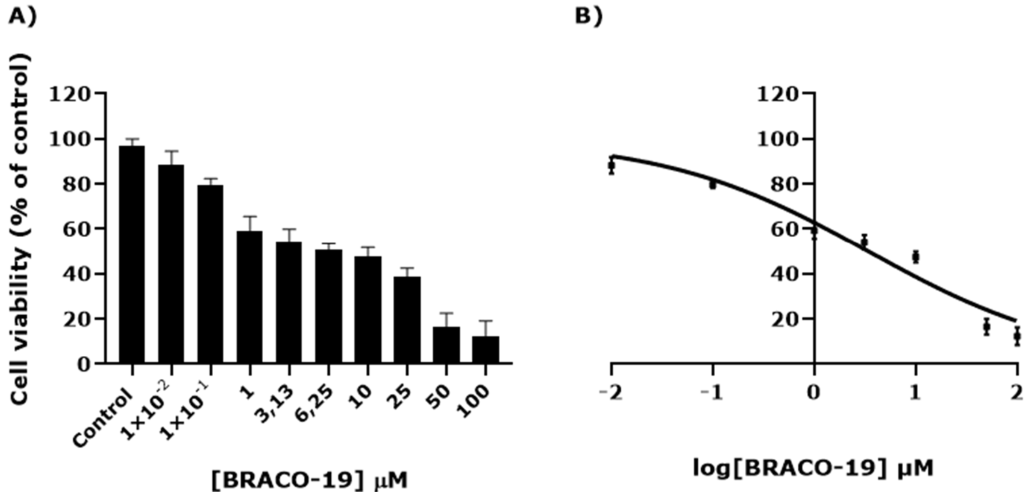

Figure S7. Cell viability after 48 h of incubation with BR[illegible]CO-19 concentrations of 1×10^{-2} to 100 µM; B) Concentration-dependent curve of cell viability (plotted using GraphPad Prism 8.0.1.).The results represent mean ± SD of at three independent experiments (n=3).

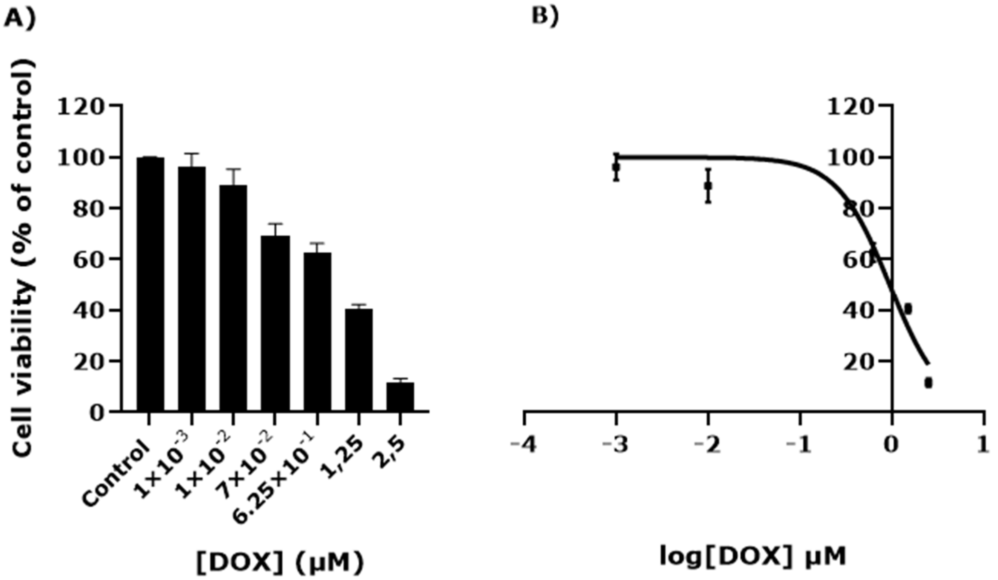

Figure S8. ([illegible]) Cell viability after 48 h of incubation with DOX concentrations in non-functionalized liposomes of 1×10^{-3} to 2.5 µM; (B) Concentration-dependent cell viability curve of DOX liposomes (plotted using GraphPad Prism 8.0.1.). The results represent mean ± SD of at three independent experiments (n=3).

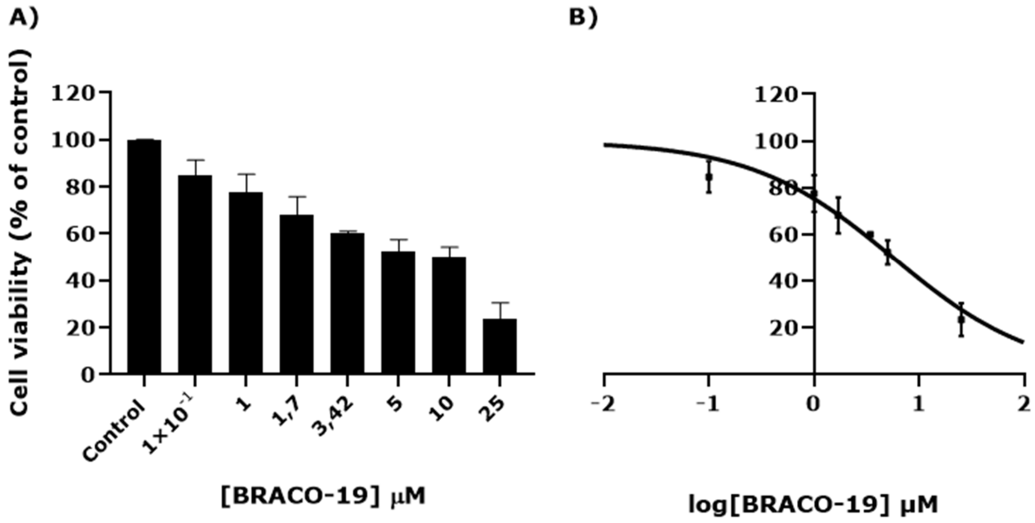

Figure S9. () Cell viability after 48 h of incubation with BRCO-19 concentrations in non-functionalized liposomes of 1 × 10^{-1} to 25 μM; (B) Concentration-dependent cell viability curve of BRCO-19 liposomes (plotted using GraphPad Prism 8.0.1.). The results represent the mean ± SD from three independent experiments (n=3).

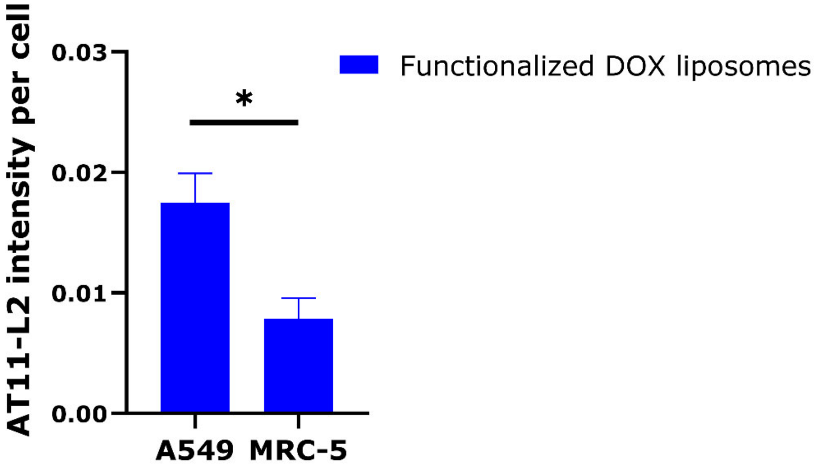

Figure S10. T11-L2 intensity per cell in the two cell lines 549 and MRC-5 after 2 h of incubation with T11-L2-DOX liposomes (plotted using GraphPad Prism 8.0.1.). The results represent the mean ± SEM from three independent experiments (n=3).* (p = 0.0314) using the unpaired two-tailed t-test.

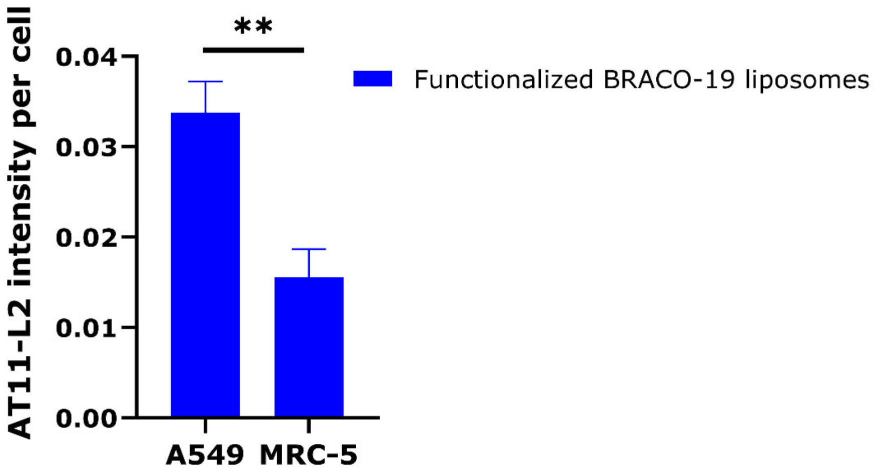

Figure S11. T11-L2 intensity per cell in the two cell lines 549 and MRC-5 after 2 h of incubation with T11-L2-BRCO-19 liposomes (plotted using GraphPad Prism 8.0.1.).The results represent the mean ± SEM from three independent experiments (n=3). ** p = 0.0064 using the unpaired two-tailed t-test.

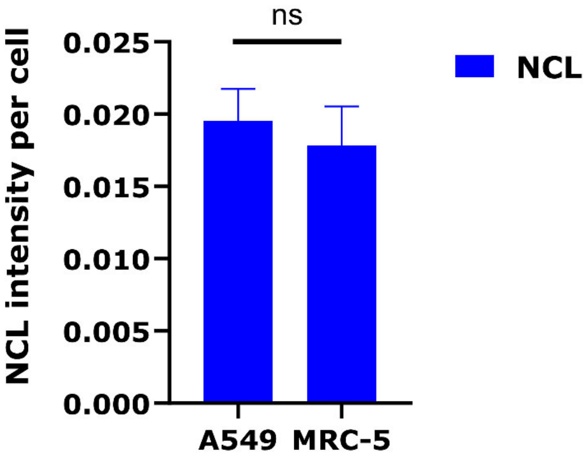

Figure S12. NCL total intensity per cell in the two cell lines 549 and MRC-5 (plotted using GraphPad Prism 8.0.1.).The results represent the mean ± SEM from three independent experiments (n=3). ns (p = 0.6256) using the unpaired two-tailed t-test.

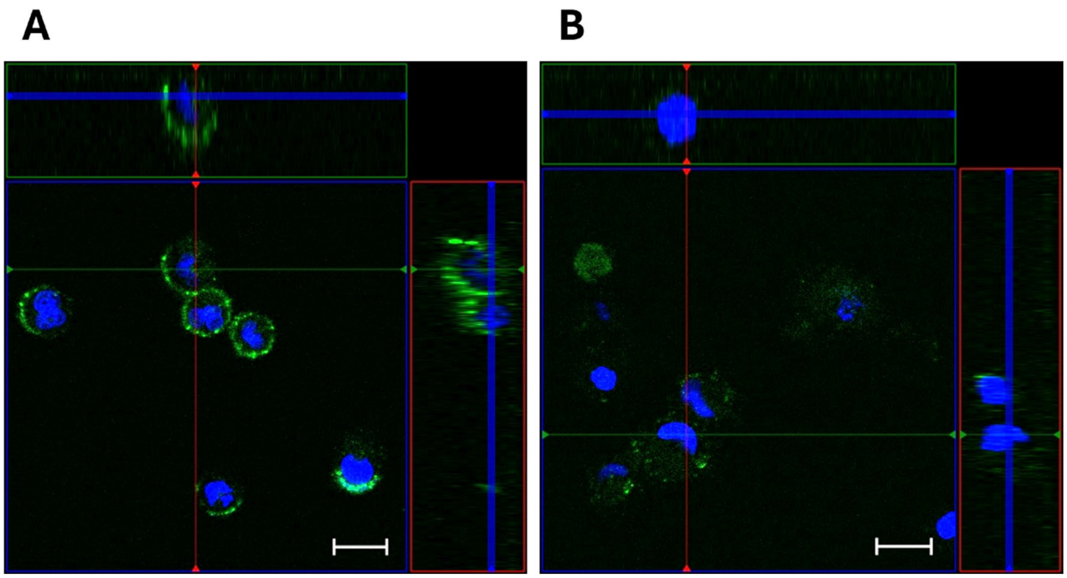

Figure S13. Representative z-stacks data showing NCL expression in (A) A549 cells and (B) in MRC5 cells. Nuclei are stained in blue (Hoechst) and NCL in green (Alexa Fluor 488®). Scale bar 20 µm.

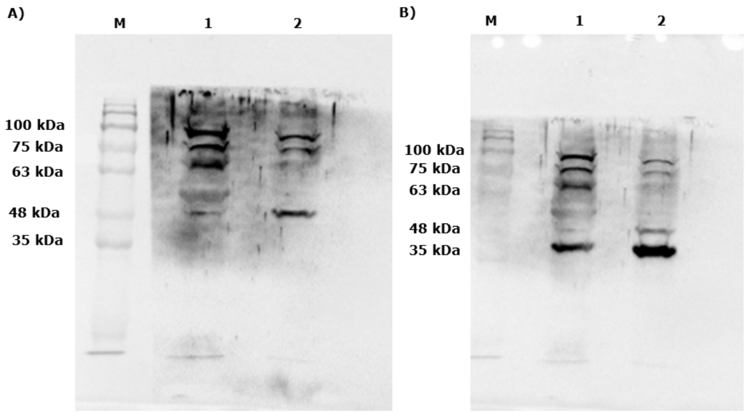

Figure S14. (A) NCL and (B) *β*-actin expression analyzed by Western Blot. Lane M – protein marker; Lane 1- A549 cells; Lane 2 – MRC-5 cells.

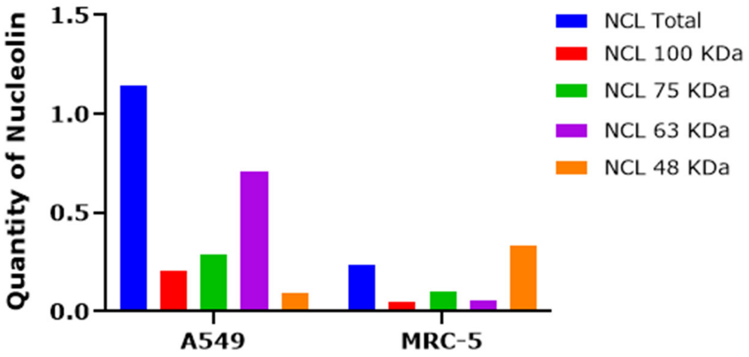

Figure S15. NCL quantification in the 48, 63, 75, and 100 kDa bands and total NCL expression (plotted using GraphPad Prism 8.0.1.).The results represent one independent experiment (n=1).
